# Nonsuicidal Self-Injury and Suicidal Behavior: A Latent Class Analysis among Young Adults

**DOI:** 10.1371/journal.pone.0059955

**Published:** 2013-03-27

**Authors:** Chloe A. Hamza, Teena Willoughby

**Affiliations:** Department of Psychology, Brock University, St. Catharines, Ontario, Canada; Federal University of Rio de Janeiro, Brazil

## Abstract

Although there is a general consensus among researchers that engagement in nonsuicidal self-injury (NSSI) is associated with increased risk for suicidal behavior, little attention has been given to whether suicidal risk varies among individuals engaging in NSSI. To identify individuals with a history of NSSI who are most at risk for suicidal behavior, we examined individual variability in both NSSI and suicidal behavior among a sample of young adults with a history of NSSI (*N* = 439, *Mage* = 19.1). Participants completed self-report measures assessing NSSI, suicidal behavior, and psychosocial adjustment (e.g., depressive symptoms, daily hassles). We conducted a latent class analysis using several characteristics of NSSI and suicidal behaviors as class indicators. Three subgroups of individuals were identified: 1) an infrequent NSSI/not high risk for suicidal behavior group, 2) a frequent NSSI/not high risk for suicidal behavior group, and 3) a frequent NSSI/high risk for suicidal behavior group. Follow-up analyses indicated that individuals in the ‘frequent NSSI/high risk for suicidal behavior’ group met the clinical-cut off score for high suicidal risk and reported significantly greater levels of suicidal ideation, attempts, and risk for future suicidal behavior as compared to the other two classes. Thus, this study is the first to identity variability in suicidal risk among individuals engaging in frequent and multiple methods of NSSI. Class 3 was also differentiated by higher levels of psychosocial impairment relative to the other two classes, as well as a comparison group of non-injuring young adults. Results underscore the importance of assessing individual differences in NSSI characteristics, as well as psychosocial impairment, when assessing risk for suicidal behavior.

## Introduction

Nonsuicidal self-injury (NSSI) refers to the direct and deliberate destruction or alteration of bodily tissue in the absence of suicidal intent [1], and includes behaviors such as self-cutting, carving, burning and hitting [2]. Among clinical inpatient samples, as many as 21% of adults [3], and 30 to 40% of adolescents engage in NSSI [4], [5]. NSSI is not only a clinical health concern, however, as recent estimates indicate that as many as 12–38% of young adults report lifetime histories of NSSI [6], [7], [8]. Although NSSI tends to have its onset in adolescence, close to 40% of individuals who engage in NSSI report first time engagement between the ages of 17 and 24 [2], [9]. Moreover, 35–72% of self-injuring young adults report current engagement in NSSI [10], [11], [12], which has led researchers to conclude that NSSI is a widely occurring health concern among university students. Although NSSI differs from suicidal behaviors on the basis of non-lethal intent [13], researchers have consistently found that young adults who engage in NSSI are at increased risk for suicidal behavior as compared to individuals who do not engage in NSSI [8], [14], [15], [16], [17], [18], [19], [20]. At the same time, however, only a minority of young adults who engage in NSSI actually engage in suicidal behavior (e.g., suicidal attempt) [19]. Given the high prevalence of NSSI among community-based samples, identifying individuals with a history of NSSI who are at risk for suicidal behavior is of critical importance to researchers, clinicians and health care providers [20]. The purpose of the present study was to identify nonsuicidal self-injurers who are most at risk for past, present and future suicidal behavior. In addition, we examined whether self-injurers with varying degrees of suicidal risk differed on several psychosocial indices.

### Assessing Risk for Suicidal Behavior

According to Joiner’s (2005) interpersonal theory of suicide, greater involvement in NSSI increases an individual’s acquired capability for suicide by habituating the individual to the fear and pain associated with taking one’s own life [21], [22]. Individuals who engage in more frequent and severe NSSI, therefore, would be expected to be at greater risk for suicidal ideation and attempts. In support of Joiner’s theory, more frequent engagement in NSSI [8], [13], [16], and greater time spent engaging in NSSI, particularly when alone [15], [22], [23], [24], have been associated with increased risk for suicidal attempts. Moreover, individuals who engage in multiple methods of NSSI (e.g., cutting, burning, etc.) are at greater risk for suicidal behavior as compared to individuals who engage in fewer methods of NSSI [22].

Recent research indicates that individuals with varying levels of engagement in NSSI may also be differentiated on the basis of psychosocial impairment. More specifically, in two studies, Klonsky and Olino [19] and Whitlock and colleagues [23] compared subgroups of individuals with varying NSSI histories on measures of psychosocial risk [19], [23]. In both of these studies, researchers identified a high risk group of self-injurers who reported frequent engagement in NSSI involving multiple methods and functions. The high risk NSSI groups were also differentiated from other NSSI groups by greater psychosocial impairment (i.e., depressive symptoms, anxiety, BPD, history of childhood abuse) and reporting of suicidal attempts.

Although the findings of Klonsky and Olino [19] and Whitlock et al. [23] indicate that individuals who engage in NSSI can be divided into subgroups depending on their NSSI characteristics (e.g., frequency), these researchers did not specifically seek to examine whether individuals with a history of NSSI could be categorized on the basis of their engagement in *both* NSSI and suicidal behavior. Similar to NSSI, there is likely to be individual variability in suicidal behavior. Grouping individuals on the basis of their engagement in both NSSI and suicidal behavior, therefore, may provide a more nuanced examination of the heterogeneity among individuals with varying histories of self-injurious behavior (e.g., frequent NSSI but low suicidal risk vs. frequent NSSI and high suicidal risk). Moreover, Klonsky and Olino and Whitlock et al. specifically examined significant group differences in lifetime suicidal ideation and attempts, but current suicidal ideation and self-reported likelihood of future suicidal attempts may also be important predictors of suicidal risk.

In the present study, we address these gaps in the literature by utilizing Latent Class Analysis (LCA) to specifically examine individual variability in both NSSI and suicidal behavior among a sample of young adults with a history of NSSI. Our objective was to identify those individuals with a history of NSSI who are most at risk for suicidal behavior. LCA is a person-centered analysis in which relationships among individuals, rather than relationships among variables, are of primary interest [25]. In addition to assessing lifetime suicidal ideation and attempts, we also included a measure of recent suicidal ideation and self-reported risk for future suicidal attempts. We expected to identify a subgroup of nonsuicidal self-injurers at high risk for suicidal behavior [21]. Moreover, we expected that self-injurers who are most at risk for suicidal behavior would be differentiated from the rest of the sample by greater psychosocial impairment.

## Methods

### Participants

The current sample was drawn from a larger sample of 1,090 (70.3% female) first-year undergraduate students (*Mage* = 19.11, *SD* = 1.05) from a mid-sized Canadian university who completed a survey about aspects of the university experience that create or reduce stress. In total, 439 respondents indicated that they had engaged in NSSI at least once and were included in the present study. Only participants who reported a history of NSSI were prompted to complete additional questions about their engagement in NSSI. Participation in this study was open to all first-year students regardless of major. In total, 87.5% of the participants were born in Canada. Consistent with the broader demographics of the region, the most common ethnic backgrounds reported other than Canadian were British (19%), Italian (16.8%), French (9.5%) and German (9%) [26]. Data on socioeconomic status indicated mean levels of education for mothers and fathers falling between “some college, university or apprenticeship program” and “completed a college/apprenticeship/technical diploma.” Furthermore, 15% of respondents lived at home with one or both parents, 9% lived off-campus with roommates, and 76% lived in campus residences. In total, less than 2% of data was missing due either to non-response or an insufficient number of responses. Missing values were imputed using the EM (expectation-maximum) algorithm. Methodological research has demonstrated that ML estimation is preferable to pair-wise deletion, list-wise deletion, or means substitution [27].

### Procedure

Students in first-year university were invited to complete a survey examining adjustment in university, by way of posters, class room announcements, website postings, and residence visits. Students could participate regardless of academic major, and were given monetary compensation ($10) or course credit for their participation. The survey was administered by trained research personal.

#### Ethics statement

The study was approved by Brock University Ethics board, and all participants provided informed written consent before participation. No minors/children were involved in the study, so no informed consent was obtained from next of kin, caretakers or guardians.

### Measures

#### Demographics

Age, sex and parental education (one item per parent), averaged for participants reporting on both parents (*r* = .40) were assessed on a scale of 1 (did not finish high school) to 6 (professional degree).

#### Nonsuicidal self-injury (NSSI)

Participants completed the Inventory of Statements about Self-Injury (ISAS) [7] to specifically address whether they had engaged in direct forms of self-injury. A list of eight self-injurious behaviors was provided (e.g., cutting, burning and head banging) and participants were asked to indicate how many times they had intentionally engaged in each of the behaviors listed, without lethal intent. Participant responses regarding lifetime frequency of NSSI were collapsed into the following six categories to create a normalized measure of NSSI frequency: 1 incident, 2–4 incidents, 5–10 incidents, 11–50 incidents, 51–100 incidents, more than 100 incidents (see [2] for a similar categorization). The Cronbach’s alpha for NSSI frequency was.77. The number of NSSI methods that participants engaged in was calculated by totalling the different types of NSSI behaviors participants endorsed. Participants were also asked to indicate whether they experienced physical pain while self-injuring (1 = no, 2 = sometimes, 3 = yes), the amount of time elapsed between the urge to self-injure and the act of NSSI (i.e., 1 = less than one hour to 6 = more than 1 day), age of most recent NSSI, and whether they self-injured alone (1 = no, 2 = sometimes, 3 = yes). The ISAS has been shown to have good internal consistency and construct validity in previous research [7], [10].

#### Suicidal behavior

Participants completed the Suicide Behaviors Questionnaire-Revised (SBQR) [28], which includes four items assessing four different dimensions of suicidality. Participants indicated: 1) whether they had ever thought about or attempted to kill themselves (i.e., lifetime suicidal ideation/attempt) on a scale from 1 (never) to 6 (I have attempted to kill myself and really hoped to die), 2) their frequency of suicidal ideation over the past 12 months (i.e., recent suicidal ideation) on a scale from 1 (never) to 5 (very often), 3) whether they had previously disclosed to anyone that they were going to attempt suicide (i.e., disclosure about suicidal behaviour) on a scale from 1 (no) to 5 (yes, more than once and really wanted to do it), and 4) their likelihood of a future suicidal attempt (i.e., future suicidal behaviour) from 1 (never) to 7 (very likely). The Cronbach’s alpha for the SBQR was.74, and the SBQR has been shown to have good internal consistency and validity in both clinical and non-clinical samples in previous research [28].

#### Well-being

Six aspects of psychological well-being were assessed including: daily hassles, difficulties with emotion regulation, depressive symptoms, self-esteem, social anxiety and behavioural inhibition. Daily hassles was assessed using 26 items in which participants were asked to indicate the frequency of being bothered by daily hassles with friends, peers, and university work (e.g., trying to get good marks) using a 3-point scale from 1 (almost never bothers me) to 3 (often bothers me). Cronbach’s alpha for the scale was.84. Participants also completed the Difficulties with Emotion Regulation Scale (DERS) [29], which included six items (e.g., when I’m upset, I have difficulty concentrating), assessed on a scale from 1 (not at all like me) to 5 (completely like me). The Cronbach’s alpha for the scale was.74. The DERS has been shown to have good internal consistency and discriminant validity among adolescents and university students [29], [30]. Depressive symptoms were measured using the Center for Epidemiological Studies Depression Scale (CES-D) [31]. Participants indicated how often they experienced 20 depressive symptoms (e.g., felt sad) from 1 (none of the time) to 5 (most of the time). The Cronbach’s alpha for the scale was.91. The CES-D has also been shown to have good internal reliability in previous research [32]. Self-esteem was assessed using Rosenberg’s (1965) 10 item scale [33], which has been widely used and shown to be internally consistent [34]. Participants were required to indicate the extent to which they agree or disagree to items such as “I take a positive attitude toward myself” using a five point scale from 1 (strongly disagree) to 5 (strongly agree). Cronbach’s alpha for the scale was.90. Social anxiety was assessed using 14 items (e.g. I feel shy around people my age that I do not know) from the Social Anxiety Scale for Children-Revised (SASC-R) [35] using a scale from 1 (almost never or never) to 4 (almost always or always). The SASC-R has been shown to have good internal reliability and validity [36], and the Cronbach’s alpha for the scale was.90. Behavioral inhibition was assessed using the Behavioral Inhibition Scale (BIS) [37]. The BIS is designed to measure participants’ sensitivity to anxiety-provoking situations (e.g., “If I think something unpleasant is going to happen I usually get pretty worked up”) using a four point scale from 1 (strongly disagree) to 4 (strongly agree). The BIS has been shown to have good internal consistency in pervious research [38]. The Cronbach’s alpha for this scale was.73.

#### Friendship quality

Friendship quality was assessed using 18 items (e.g., my friends accept me as I am) from Armsden and Greenberg’s (1987) Inventory of Parent and Peer Attachment [39], using a scale from 1 (almost never or never) to 4 (almost always or always). Cronbach’s alpha was.89.

#### Parental relationship

Participants completed 17 items (e.g., “I trust my mother”) from the Inventory of Parent and Peer Attachment [39] for both parents using a four point scale from 1 (almost never or never) to 4 (almost always or always). A parental attachment score was calculated by averaging scores from both parents (*r* = .48). Cronbach’s alpha for the scale was.91. Participants also completed the Psychological Control Scale [40] for both parents (i.e., “my father is a person who changes the subject whenever I have something to say”) using a three point scale from 1 (not at all like him) to 3 (a lot like him). Scores for both parents were averaged into a parental psychological control score (*r* = .37). Cronbach’s alpha for this scale was 0.83. Finally, participants completed the parental criticism subscale from the Multidimensional Perfectionism Scale [41] which included items such as, “My parents never try to understand my mistakes” using a three point scale from 1 (strongly disagree) to 4 (strongly agree). Cronbach’s alpha for this scale was.82.

#### Delinquency

Delinquency was measured with five items assessing stealing money from parents/roommates, shoplifting, destroying other people’s property, impaired driving, or been the passenger in a vehicle with a driver who was impaired [42]. A composite score was created with higher scores indicating greater delinquency. Cronbachs alpha was.68. High reliability would not be expected given that an individual engaging frequently in one type of delinquent behavior may not necessarily engage frequently in other types of delinquent behaviors [43].

### Plan of Analysis

Latent class analysis (LCA) was conducted using Mplus, Version 6.1 [44] to explore subgroup heterogeneity among individuals engaging in NSSI. Latent class indicators included the NSSI variables (e.g., lifetime frequency, most recent engagement), as well as the suicidal behavior variables (e.g., lifetime suicidal ideation and suicidal attempts). In order to determine the number of groups that were best represented by the data, four criteria were considered: 1) Bayesian information criterion (BIC), such that smaller values of BIC indicate a better fit model, 2) significance of the Lo-Mendell-Rubin Adjusted Likelihood Ratio Test (LMR-LRT), such that once non-significance is reached, the number of classes prior to non-significance are defined as the appropriate number, 3) no classes contain less than 5% of the total sample, and 4) that entropy (an index of confidence that individuals belong to the correct class and that adequate separation between latent classes exist) is greater than.80 [45]. Following the latent class analysis, one-way ANOVAs and post-hoc follow-up testing (i.e., Tukey) were conducted to compare individuals within each class across demographic characteristics (i.e., age, sex, parental education) and the psychosocial indices (i.e., daily hassles, difficulties with emotion regulation, depressive symptoms, self-esteem, social anxiety, behavioral inhibition, delinquency, friendship quality, parental attachment, parental psychological control and parental criticism).

## Results

### Preliminary Analyses

Overall, among our sample of 439 self-injuring young adults, 5.9% of participants had engaged in NSSI once, 15.8% engaged in the behavior 2–4 times, 24% engaged in the behavior 5–10 times, 33.0% engaged in the behavior 11–50 times, 7.1% engaged in the behavior 51–100 times and 14.2% engaged in the behavior more than 100 times. The most commonly endorsed types of self-injury were self-hitting and head banging (21.9%), hair pulling and pinching (24%), and cutting (12.1%). In total, 30.7% reported using only one method of NSSI, 28.8% reported two methods of NSSI, 17.4% reported three methods, 10% reported four methods, and 13.1% reported five or more methods of NSSI.

### Primary Analyses

#### Extraction of latent classes

Latent class analyses were conducted for 1–4 class solutions, and the best-fitting solution was three classes (see Table 1). The three class model had a lower BIC value relative to the other classes, and an entropy value greater than 0.80. In addition, the three class solution had no classes less than 5%. Furthermore, the LMR-LRT was significant, which indicated that the three class solution provided a better fit to the data than the two class solution. In contrast, the LMR-LRT for the four class solution was non-significant, suggesting the three class solution provided the better fit to the data than the four class solution. Results indicated that 67.7% of participants belonged to Class 1 (“low frequency NSSI/not high risk for suicidal behavior”). Individuals in Class 1 were characterized by lower frequency engagement in NSSI, less recent NSSI, and fewer methods of NSSI than the other two classes. Individuals in Class 1 also had lower levels of lifetime suicidal ideation/attempts, less recent suicidal ideation, and less likelihood of future attempt as compared to Class 3 (See Figure S1). In contrast, individuals in Class 2 (“high frequency NSSI/not high risk for suicidal behavior) (19.8%) reported higher frequency of engagement in NSSI, more recent NSSI, and more methods of NSSI as compared to Class 1. Individuals in Class 2, however, similarly reported lower levels of lifetime suicidal ideation/attempts, lower recent suicidal ideation and lower likelihood of future suicidal attempts as compared to Class 3. Finally, individuals in Class 3 (“high frequency NSSI/high risk for suicidal behavior”) (12.5%) reported higher frequency of engagement in NSSI, more recent NSSI and more methods of NSSI than Class 1. Class 3 also reported higher levels of lifetime suicidal ideation/attempts, higher recent suicidal ideation, and greater risk for future suicidal attempts as compared to Class 1 and Class 2. Class 3 was also the only group that met the clinical cut-off for high suicide risk on the SBQ-R, which is why this group was labeled the high risk for suicidal behavior group. To ensure classes were classified appropriately, one-way ANOVAs were conducted using class membership as the independent variable and each of the class indicators as dependent variables. Results supported our class characterizations, and significant group differences are presented in Table 2.

**Table 1:** Fit Indices for Latent Class Analysis.

	1	2	3	4
BIC	13900.997	13419.045	13312.442	13256.482
Entropy	–	0.950	0.838	0.837
Class >5%	No	No	No	No
LMR-LRT	–	Sig	Sig	NS

Note*:* BIC = Bayesian information criterion (smaller values indicate better model fit). Entropy at least.80 (higher values indicates well identified classes). Class >5% (any class smaller than 5% not sufficient). LMR-LRT = Lo-Mendell-Rubin Adjusted Likelihood Ratio Test, test of fit between the model of interest (e.g., three-class model) and the model with one less class (e.g., two-class model). Sig = significant. NS = non-significant.

**Table 2:** Significant differences among classes – means and standard deviations.

	DF1	DF2	F	*p*	η^2^	Class 1 (N = 297)	Class 2 (N = 87)	Class 3 (N = 55)
**Characteristics of Latent Classes**								
Lifetime frequency of NSSI	2	436	90.34	***	.37	3.04 (1.12)^a^	5.10(0.95)^c^	4.29 (1.10)^b^
Age of most recent NSSI	2	436	8.54	***	.05	16.24 (2.08)^a^	17.21 (1.86)^a,b^	17.82 (1.89)^b^
Pain during NSSI	2	436	7.32	.001	–	2.05 (0.71)^a^	2.31 (0.65)^b^	2.23 (0.74)^b^
Time elapsed NSSI	2	436	1.44	.348	–	2.19 (1.64)^a^	2.22 (1.83)^a^	1.86 (1.36)^a^
Number of methods of NSSI	2	436	139.33	***	.48	1.78 (0.82)^a^	4.40 (1.44)^c^	3.65 (1.94)^b^
Alone during NSSI	2	436	5.31	.005	–	2.27 (0.77)^a^	2.38 (0.68)^a^	2.69 (0.53)^b^
Lifetime suicidal ideation/attempts	2	436	71.89	***	.25	1.62 (0.89)^a^	2.33 (1.34)^b^	3.53 (1.46)^c^
Past year suicidal ideation	2	436	255.48	***	.54	1.27 (0.59)^a^	1.37 (0.59)^a^	3.58 (1.97)^b^
Disclosure about suicide	2	436	19.89	***	.08	1.28 (0.63)^a^	1.70 (1.01)^b^	1.93 (1.08)^b^
Future suicide attempt	2	436	217.16	***	.50	1.31 (0.64)^a^	1.56 (0.76)^a^	3.62 (1.16)^b^
Total SBQ-R score	2	436	257.50	***	.62	4.40(1.61)^ a^	5.56(1.71)^ b^	10.74(1.59)^c^
**Psychosocial indices**								
Daily hassles	2	436	13.37	***	.06	1.93 (0.30)^a^	1.99 (0.29)^a^	2.16 (0.29)^b^
Difficulties with emotion regulation	2	436	14.44	***	.06	2.85 (0.71)^a^	2.89 (0.82)^a^	3.43 (0.66)^b^
Depressive symptoms	2	436	43.84	***	.20	2.14 (0.62)^a^	2.28 (0.59)^a^	2.99 (0.67)^b^
Self-esteem	2	436	39.72	***	.15	3.80 (0.67)^b^	3.58 (0.67)^b^	2.95 (0.60)^a^
Social anxiety	2	436	20.47	***	.09	1.74 (0.52)^a^	1.83 (0.51)^a^	2.24 (0.61)^b^
Behavioral inhibition	2	436	13.37	***	.06	2.76 (0.44)^a^	2.81 (0.45)^a^	3.09 (0.38)^b^
Delinquency	2	436	1.13	.323	–	1.48 (0.57)^a^	1.58 (0.58)^a^	1.55 (0.55)^a^
Friendship quality	2	436	11.56	***	.05	3.19 (0.48)^b^	3.09 (0.49)^b^	2.85 (0.42)^a^
Parental attachment	2	436	18.72	***	.08	2.87 (0.43)^c^	2.68 (0.47)^b^	2.52 (0.39)^a^
Parental criticism	2	436	6.88	***	.03	2.11 (0.71)^a^	2.29 (0.77)^a,b^	2.48 (0.74)^b^
Parental psychological control	2	436	9.95	***	.04	1.48 (0.36)^a^	1.67 (0.44)^b^	1.64 (0.39)^b^

Note. Means in the same row with different superscripts are significantly different at *p*<.001. Higher scores indicate greater frequency of engagement in NSSI, age of most recent NSSI, pain during NSSI, time elapsed between urge to self-injure and act of NSSI, number of methods of NSSI, being alone during NSSI, more lifetime suicidal ideation/attempts, greater past year suicidal ideation, greater disclosure about suicidal behavior, and more likely to make a future suicidal attempt. Higher scores indicate greater daily hassles, difficulties with emotion regulation, depressive symptoms, self-esteem, social anxiety, behavioral inhibition, delinquency, friendship quality, parental attachment, parental criticism and parental psychological control. Scores on the SBQR range from 3–18, with a clinical cutoff score of 7. ***p<0.001.

#### Differences among classes on psychosocial indices

Given the use of multiple ANOVAs, a reduced alpha of *p*<0.001 was used to establish significant differences among groups. Results indicated that groups did not significantly differ across age, gender, or parental education, all *p*s >.001. Significant group differences were found across psychosocial indices, including daily hassles, difficulties with emotion regulation, depressive symptoms, self-esteem, social anxiety, behavioral inhibition, friendship quality, parental attachment, parental criticism, and parental psychological control. Groups did not significantly differ on delinquency. Follow-up Tukey analyses revealed that overall, Class 3 reported the highest levels of risk across psychosocial indices as compared to Class 1 and Class 2 (see Table 2). Although Class 1 and 2 did not significantly differ across many of the psychosocial indices, Class 2 reported significantly lower levels of parental attachment and higher levels of parental psychological control as compared to Class 1.

#### Non-NSSI group comparison

One issue that the above analyses did not address, was whether the three classes differed on measures of psychosocial risk compared to a non-injuring group. In order to test this issue, we examined whether Class 1, Class 2, and Class 3 significantly differed from a comparison group of a random sample of 250 participants without a history of NSSI taken from the larger sample (see Table 3). A random subset of 250 participants was used to ensure that the comparison group was not disproportionately larger than the other groups. The ANOVA analyses and Tukey follow-up comparisons revealed that all the groups did not significantly differ on age, sex or parental education (all *p*s >0.001). Importantly, Class 1 did not significantly differ from the comparison group on any of the psychosocial indices. In contrast, Class 2 reported significantly greater psychosocial risk than the comparison group on several psychosocial indices (i.e., depressive symptoms, delinquency, parental psychological control, suicidal behavior, self-esteem, friendship quality, and parental attachment). Finally, Class 3 reported significantly higher risk than the comparison group across *all* of the psychosocial indices.

**Table 3:** Class comparisons to a group of non-injurers.

Psychosocial risk factor	Control (N = 250)	Class 1 (N = 298)	Class 2 (N = 86)	Class 3 (N = 55)
Daily hassles	1.91 (0.33)^a^	1.93 (0.30)^a^	1.99 (0.29)^a^	2.16 (0.29)^b^
Difficulties with emotion regulation	2.69 (0.76)^a^	2.85 (0.71)^a^	2.89 (0.82)^a^	3.43 (0.66)^b^
Depressive symptoms	2.01 (0.63)^a^	2.14 (0.62)^a,b^	2.28 (0.59)^b^	2.99 (0.67)^c^
Self-esteem	3.91 (0.67)^c^	3.80 (0.67)^c,b^	3.58 (0.67)^b^	2.95 (0.60)^a^
Social anxiety	1.68 (0.51)^a^	1.74 (0.52)^a^	1.83 (0.51)^a^	2.24 (0.61)^b^
Behavioral inhibition	2.72 (0.48)^a^	2.76 (0.44)^a^	2.81 (0.45)^a^	3.09 (0.38)^b^
Delinquency	1.34 (0.51)^a^	1.48 (0.57)^a,b^	1.58 (0.58)^b^	1.55 (0.55)^b^
Friendship quality	3.29 (0.47)^c^	3.19 (0.48)^c,b^	3.09 (0.49)^b^	2.85 (0.42)^a^
Parental attachment	2.91(0.44)^c^	2.87 (0.43)^c^	2.68 (0.47)^b^	2.52 (0.39)^a^
Parental criticism	2.03 (0.74)^a^	2.11 (0.71)^a^	2.29 (0.77)^a,b^	2.48 (0.74)^b^
Parental psychological control	1.40 (0.34)^a^	1.48 (0.36)^a^	1.67 (0.44)^b^	1.64 (0.39)^b^
Lifetime suicidal ideation/attempts	1.40 (0.82)^a^	1.62 (0.89)^a^	2.33 (1.34)^b^	3.53 (1.46)^c^
Past year suicidal ideation	1.25 (0.68)^a^	1.27 (0.59)^a^	1.37 (0.59)^a^	3.58 (1.97)^b^
Disclosure about suicide	1.11 (0.44)^a^	1.28 (0.63)^a^	1.70 (1.01)^b^	1.93 (1.08)^b^
Future attempt	1.24 (0.69)^a^	1.31 (0.64)^a,b^	1.56 (0.76)^b^	3.62 (1.16)^c^

Note: Means in the same row with different superscripts are significantly different at *p*<.001. Higher scores indicate greater daily hassles, greater difficulties with emotion regulation, greater depressive symptoms, higher self-esteem, greater social anxiety, greater behavioral inhibition, greater delinquency, greater friendship quality, greater parental attachment, greater parental criticism, greater parental psychological control, more lifetime suicidal ideation/attempts, greater past year suicidal ideation, greater disclosure about suicidal behavior, and more likely to make a future suicidal attempt.

## Discussion

Despite increased research on the association between NSSI and suicidal behavior [18], little research has examined individual variability in suicidal risk among individuals engaging in NSSI. To identify nonsuicidal self-injurers who are most at risk for suicidal behaviour, we conducted a person-centered analysis (i.e., LCA) among a sample of young adults with a history of NSSI. Results of the LCA revealed three distinct subgroups of individuals with varying presentations of NSSI and suicidal behavior. The three subgroups differed not only with respect to their patterns of self-injurious behaviors, but they also could be discriminated on measures of psychosocial risk. These findings offer clinicians with new insight into who may be most at risk for suicidal behaviour among nonsuicidal self-injurers, and can serve to inform intervention and prevention programming aimed at reducing suicidal risk among individuals with a history of NSSI.

The first subgroup we identified consisted of 68% of young adults with a history of NSSI (i.e., Class 1). Individuals in this group were characterized by lower frequency engagement in NSSI, and fewer methods of NSSI than the other two groups (i.e., Class 2 and 3). Importantly, individuals in our “low frequency NSSI/not at high risk for suicidal behavior” group were also characterized by lower levels of suicidal behavior compared to the other two classes. Moreover, these individuals did not report higher levels of suicidal behavior as compared to the comparison group of non-injuring participants. Thus, although 39.5% of the larger sample reported a history of NSSI, the results of the present study indicate that the majority of young adults who engage in NSSI do so infrequently and are not at elevated risk for suicidal behavior. Thus, future research on the link between NSSI and suicidal behavior should take into account variability among individuals engaging in NSSI.

The other two classes of self-injurers consisted of individuals who engaged in more frequent NSSI, recent NSSI, and more methods of NSSI than Class 1. Importantly, although these individuals shared similar NSSI characteristics, they differed on the basis of their engagement in suicidal behavior. Our study is the first, therefore, to identify variability in suicidal risk among individuals engaging in frequent and multiple methods of NSSI. Our findings suggest that assessing the frequency, number of methods, or age of most recent NSSI, may not be sufficient to identify individuals most at risk for suicidal behavior. In fact, although Class 2 reported the most frequent engagement in NSSI, as well as the most methods of NSSI, these individuals reported significantly lower levels of engagement in suicidal behavior as compared to Class 3. Only by conducting a person-centered analysis using both NSSI and suicidal behavior were we able to identify these two distinct groups.

Interestingly, social context (i.e., whether NSSI occurred alone) was the only NSSI characteristic to differentiate individuals in Classes 2 and 3. Our findings are consistent with work by Glenn and Klonsky [15], and suggest that the extent to which self-injury occurs alone is an important and easily accessible marker of suicidal risk among self-injurers. Moreover, our findings provide new insight in the conditions under which NSSI may lead to suicidal behavior. Specifically, individuals who engaged in frequent NSSI and suicidal behavior were also most likely to report *current* suicidal ideation. When assessing risk for suicidal behavior among self-injurers, therefore, the assessment of suicidal ideation within the past year may be a more important predictor of suicidal risk than NSSI history.

In a secondary analysis, we compared our three classes of nonsuicidal self-injurers to a comparison group of non-injuring young adults. Importantly, the majority of the self-injurers (i.e., Class 1) did not significantly differ from the comparison group of non-injurers on measures of psychosocial risk. In contrast, individuals in Classes 2 and 3 were at greater risk for psychosocial impairment as compared to the group of non-injuring individuals, although Class 3 reported greater psychosocial distress than Class 2. It is important to note, however, that only individuals in Class 3 met the clinical cut-off for high suicidal risk on the SBQ-R. Recall that according to Joiner [21], individuals who engage in NSSI on a frequent basis may be at increased risk for suicidal behavior because they habituate to the fear and pain associated with taking one’s own life (i.e., acquired capability for suicide) [22]. Another tenant of Joiner’s theory, however, is that only individuals who experience suicidal desire (i.e., perceived burdensomeness and thwarted belongingness) and have acquired capability for suicide, will actually make a suicidal attempt [46]. Therefore, it may be that individuals in Class 3 were at greatest risk for suicidal behavior engagement because they engaged in highly frequent NSSI (i.e., higher levels of acquired capability for suicide), as well as experienced high levels of psychosocial distress (i.e., greater risk for suicidal desire) and suicidal ideation. Our findings highlight the importance of assessing NSSI history, in combination with psychosocial impairment, to identify those individuals most at risk for suicidal behavior.

An unexpected finding that is important to highlight, however, is that individuals in Classes 2 and 3 reported significantly greater pain during NSSI than Class 1. Recall that Nock and colleagues [22] found that no pain during NSSI was associated with increased risk for suicidal behavior, which is more consistent with Joiner’s theory that NSSI habituates an individual to the pain associated with taking one’s own life [21], [46]. It may be, however, that individuals who have become desensitized to the pain during NSSI (i.e., frequent engagers in NSSI), increase the frequency and number of methods used during NSSI to increase painful experiences. Indeed, two commonly endorsed motivations for engaging in NSSI are anti-dissociation (i.e., to reduce feelings of numbness) and feeling generation (i.e., to feel something, even if it is pain) [7]. If NSSI does lead to decreased sensitivity to pain over time, then individuals may have to increase their frequency of engagement in NSSI to produce the desired experience of pain.

### Limitations

Despite the many strengths of our study, including the use of a large sample, our unique attempt to assess subgroups of self-injurers, as well as the assessment of several characteristics of NSSI and suicidal behavior, our study is not without limitations. First, given the concurrent design of the present study, we cannot be certain about the directionality of effects. Although theory [21], recent research [18], and longitudinal findings [14], [17] indicate that NSSI increases risk for suicidal behavior (and not vise versa), we did not directly test bidirectional associations among NSSI and suicidal behavior. It may be that suicidal behavior, therefore, also increases risk for nonsuicidal self-injury. Moreover, although we found several psychosocial indices that differentiated our three subgroups, it is unclear whether individuals in each of the subgroups reported greater risk prior to their engagement in self-injurious behavior, or as a result of their engagement in self-injurious behaviors. Only longitudinal research can specifically address whether the psychosocial indices we assessed preceded the development of self-injurious behavior. Nevertheless, our findings provide clinicians with several measures of psychosocial risk that can be used to discriminate self-injurers at high risk for current and future suicidal behavior.

Secondly, although the present sample included a large sample representative of a particular university in Canada, the majority of the participants enrolled in the study were of western descent and born in Canada; therefore, our findings may not generalize to other geographic regions, including those with differing ethnic and/or demographic backgrounds. Furthermore, our study specifically sampled first-year university students and therefore may not be generalizable to the wider student population (i.e., upper year students) or young adults not attending university. It should also be noted that previous research has found that clinical samples report greater co-occurrence of NSSI and suicidal behavior as compared to community-based samples [14]; therefore, the latent class analysis we applied may yield different results among a clinical sample. Regardless, research has shown that first year university may represent a period of increased NSSI initiation as well as increased risk for suicidal ideation [2], [23], so understanding risk for NSSI and suicidal behavior during this time period is important to clinicians and school-based counselors in the areas of risk assessment and intervention.

Third, our study required participants to recall their lifetime engagement in NSSI and suicidal behavior, so it is possible that our study is subject to recall errors. Importantly, in addition to assessing lifetime NSSI and suicidal behavior, we also tried to incorporate assessments of more recent self-injurious behavior engagement, by including age of most recent NSSI, as well as past year suicidal ideation. Regardless, it would be useful for future research to assess frequency of NSSI and suicidal behavior in real time, using ecological moments sampling, such as the use of daily diaries. Reporting on multiple incidents of NSSI and behaviors would also provide an opportunity to assess the characteristics of multiple episodes of self-injurious behaviors.

### Conclusions

In the present study, we sought to identify non-suicidal self-injurers who are most at risk for suicidal behavior. Importantly, we found that the majority of young adults who engaged in NSSI did so infrequently, and did not engage in suicidal behaviors (i.e., no greater risk than a comparison group of non-injurers). Among the minority of young adults who engaged in more frequent NSSI, recent NSSI, and multiple methods of NSSI, we identified two distinct subgroups of individuals (i.e., Class 2 and Class 3). Individuals in Class 3 met the clinical cut-off score for high risk for suicidal behavior on the SBQ-R, and were differentiated from the other classes by greater frequency of being alone when self-injuring, and higher levels of psychosocial impairment. To identify individuals with a history of NSSI who are most at risk for suicidal behavior, therefore, clinicians should assess NSSI frequency and scores on the SBQ-R, particularly degree of current suicidal ideation. Moreover, clinicians should also inquire about the social context in which NSSI occurs, and the extent to which individuals are experiencing psychosocial impairment (e.g., depressive symptoms, anxiety) relative to a comparison group of non-injurers.

## Supporting Information

Figure S1 Standardized means of latent classes on class indicators. Note: Higher scores indicate higher frequency of engagement in NSSI, more recent NSSI, greater pain during NSSI, greater time elapsed between urge to self-injure and act of NSSI, greater number of methods of NSSI, more likely to be alone when engaging in NSSI, more lifetime suicidal ideation/attempts, greater past year suicidal ideation, greater disclosure about suicidal behavior, and more likely to make a future suicidal attempt.
(TIF)
