# Supplementary figures and images for: Nonsuicidal Self-Injury and Suicidal Behavior: A Latent Class Analysis among Young Adults
Source: PLoS One. 2013 Mar 27;8(3):e59955. doi: 10.1371/journal.pone.0059955 (PMC3609776; doi:10.1371/journal.pone.0059955)

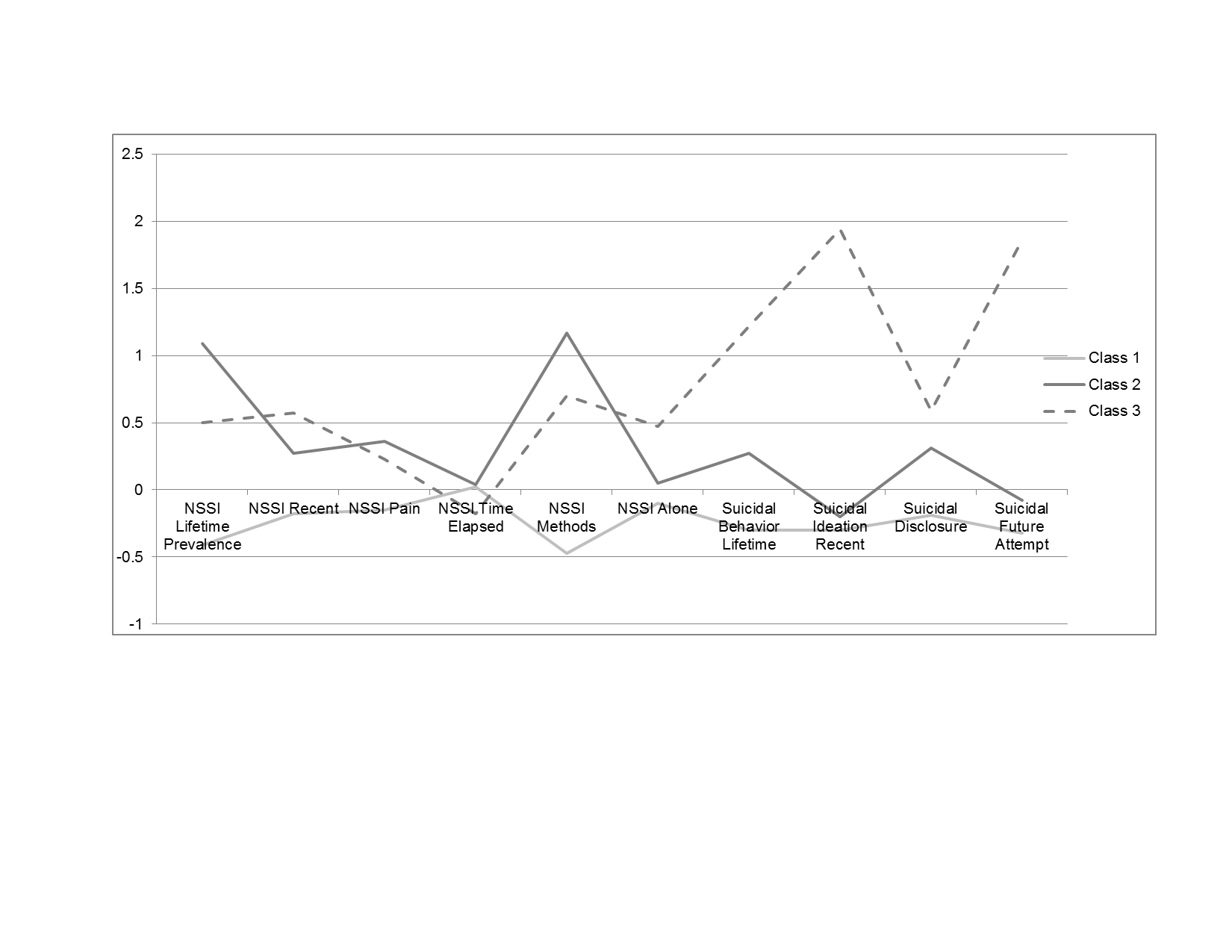

Supplement: Figure S1 — Standardized means of latent classes on class indicators. Note: Higher scores indicate higher frequency of engagement in NSSI, more recent NSSI, greater pain during NSSI, greater time elapsed between urge to self-injure and act of NSSI, greater number of methods of NSSI, more likely to be alone when engaging in NSSI, more lifetime suicidal ideation/attempts, greater past year suicidal ideation, greater disclosure about suicidal behavior, and more likely to make a future suicidal attempt. (TIF) [file pone.0059955.s001.tif]
